# Clinical value of a screening tool for tumor predisposition syndromes in childhood cancer patients (TuPS): a prospective, observational, multi-center study

**DOI:** 10.1007/s10689-021-00237-1

**Published:** 2021-03-09

**Authors:** Floor A. M. Postema, Saskia M. J. Hopman, Corianne A. J. M. de Borgie, Cora M. Aalfs, Jakob K. Anninga, Lieke P. V. Berger, Fonnet E. Bleeker, Charlotte J. Dommering, Natasha K. A. van Eijkelenburg, Peter Hammond, Marry M. van den Heuvel-Eibrink, Janna A. Hol, Wijnanda A. Kors, Tom G. W. Letteboer, Jan L. C. M. Loeffen, Lisethe Meijer, Maran J. W. Olderode-Berends, Anja Wagner, Raoul C. Hennekam, Johannes H. M. Merks

**Affiliations:** 1 Department of Pediatrics, Emma Children’s Hospital, Amsterdam UMC, University of Amsterdam, Amsterdam, The Netherlands; 2 Princess Máxima Center for Pediatric Oncology, Utrecht, The Netherlands; 3 Department of Genetics, University Medical Center Utrecht, Utrecht, The Netherlands; 4 Clinical Research Unit, Amsterdam UMC, University of Amsterdam, Amsterdam, The Netherlands; 5 Department of Genetics, University Medical Center Groningen, University of Groningen, Groningen, The Netherlands; 6 Department of Clinical Genetics, Netherlands Cancer Institute, Amsterdam, The Netherlands; 7 Department of Clinical Genetics, Amsterdam UMC, Vrije Universiteit Amsterdam, Amsterdam, The Netherlands; 8 Nuffield Department of Obstetrics and Gynecology, University of Oxford, Oxford, UK; 9 Department of Clinical Genetics, Erasmus Medical Center, Rotterdam, The Netherlands

**Keywords:** Pediatrics, Imaging, Three-dimensional, Genetic predisposition to disease, Neoplasms, Genetic screening

## Abstract

Recognizing a tumor predisposition syndrome (TPS) in a child with cancer is of clinical relevance. Earlier we developed a screening tool to increase diagnostic accuracy and clinical efficiency of identifying TPSs in children with cancer. Here we report on the value of this tool in clinical practice. TuPS is a prospective, observational, multi-center study including children newly diagnosed with cancer from 2016 to 2019 in the Netherlands. Children in whom a TPS had been diagnosed before the cancer diagnosis were excluded. The screening tool consists of a checklist, 2D and 3D photographic series and digital assessment of these by a clinical geneticist. If a TPS was suspected, the patient was assessed positive and referred for routine genetic consultation. Primary aim was to assess the clinical value of this new screening tool. Of the 363 included patients, 57% (208/363) were assessed positive. In 15% of patients (32/208), the 2D photographic series with (n = 12) or without (n = 20) 3D photographs were decisive in the positive assessment. In 2% (4/208) of positive assessed patients, a TPS was diagnosed, and in an additional 2% (4/208) a germline variant of uncertain significance was found. Thirty-five negatively assessed patients were evaluated through routine genetic consultation as controls, in none a TPS was detected. Using the screening tool, 57% of the patients were assessed as suspected for having a TPS. No false negative results were identified in the negative control group in the clinical care setting. The observed prevalence of TPS was lower than expected, due to selection bias in the cohort.

**Supplementary Information:**

The online version contains supplementary material available at 10.1007/s10689-021-00237-1.

## Introduction

It has been reported that ~ 7–10% of children with cancer has a tumor predisposition syndrome (TPS) [1–5]. Recognizing TPSs is of major clinical relevance but can be difficult [1, 6–9], urging all children with cancer to be systematically assessed for the possibility of having a TPS [1, 10]. Currently, it is mainly the treating pediatric oncologist who decides if a child will or will not be referred for clinical genetic consultation. Although molecular genetic assessments are increasingly important in the diagnostic process of TPSs, the gold standard is still considered to be the assessment by a clinical geneticist (CG) [11]. This assessment is based on the total of data obtained from the patient, the tumor and the family, and based on this information the CG decides whether or not molecular analyses should be performed. There are several reasons for this. First, in a marked number of TPSs no molecular defect is known at the present and the diagnosis has to remain clinical. Second, finding a variant in a gene in a molecular study is not proving that this variant is also causative for the tumor of the patient. It needs careful evaluation of all data, both clinical and molecular, to decide whether a causative association is likely or proven. Furthermore, panel sequencing will not detect several other mechanisms that explain a genetic cause, such as a methylation defect or position effects of genes. It will take several decades before our knowledge and experience and technical abilities have increased in such a way the mere availability of molecular genetic data is sufficient to indicate a causal relationship. Lastly, in many countries in the world molecular studies are available in only a very limited manner, which asks for a very stringent screening of patients to be studied molecularly. Therefore, in the present study a central role of the CG as gold standard was chosen.

A standardized screening tool can optimize systematic evaluation of all children with cancer for a TPS and guide referral to genetics. This would enhance a more efficient use of clinical genetic care for children with cancer. The screening should be based on (1) the type and number of tumors, (2) relevant medical history, (3) cancer history in patient and/or family, and (4) morphological abnormalities [7–9, 12–14].

We developed such a screening tool, consisting of a standardized childhood cancer syndrome checklist (CCSC), 2D and 3D photographic series, and digital assessment of all three elements by a CG [13, 15]. The primary aim of the present study was to prospectively assess the clinical value of this screening tool in a cohort of children with cancer in whom no syndrome had been diagnosed before the tumor was detected.

## Materials and methods

### Study design and setting

**Fig. 1:**
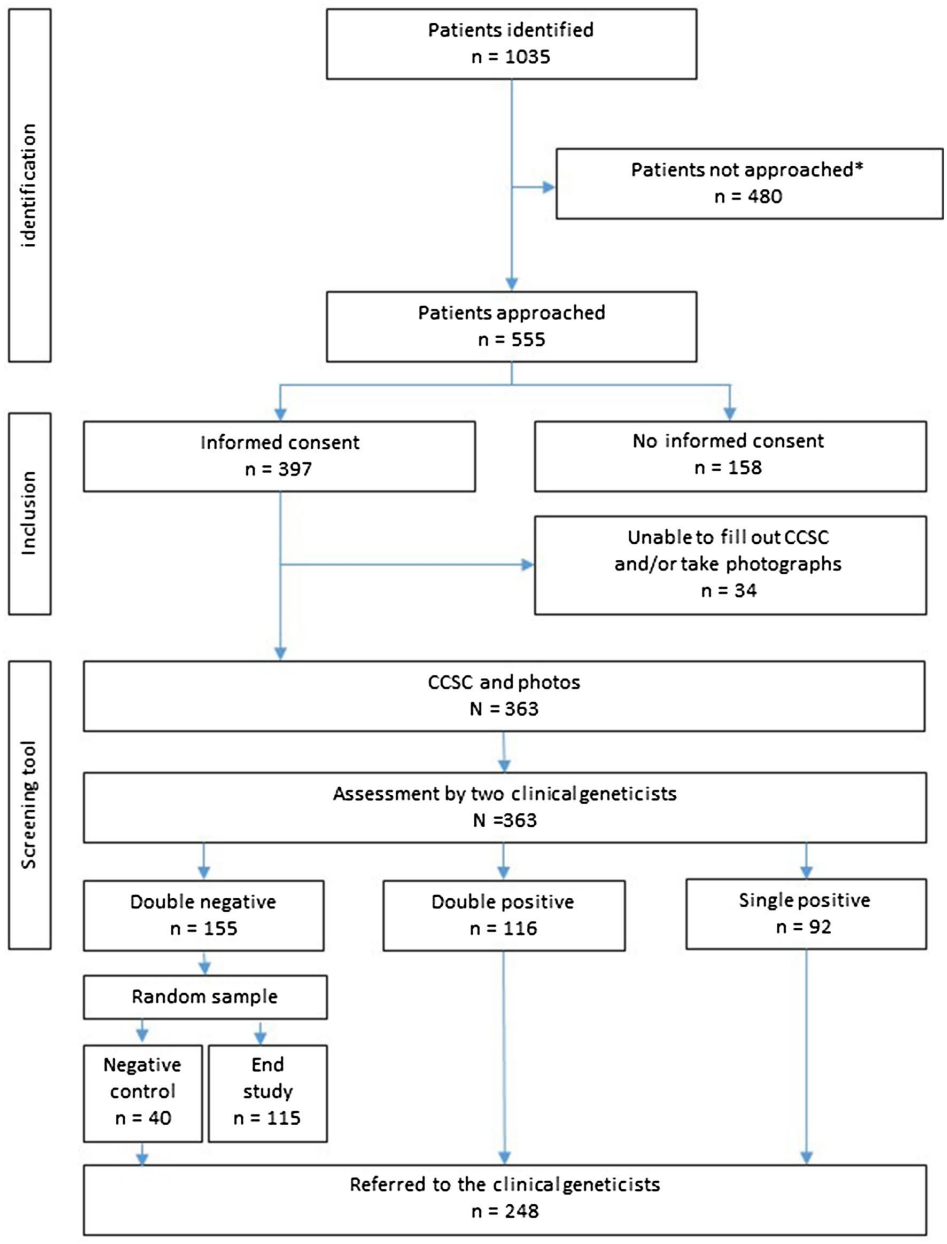
Flowchart of inclusions of the study participants. Asterisk represents probably due to: changing organization of pediatric oncology care in the Netherlands; fulfilling exclusion criteria; patient too ill to be included; referral interfering with treatment schedules; miscellaneous other reasons

This study was a prospective, observational, nationwide, multicenter cohort study, which involved all pediatric oncology centers (n = 6) and their allied clinical genetic departments in the Netherlands [15]. As of June 2018, all care for Dutch children with cancer has been centralized in a single national center (Princess Máxima Center for Pediatric Oncology). From that moment onwards, inclusion took only place in this center. The study was approved by The Medical Ethical Committee of the Amsterdam University Medical Center (W14_251 #14.17.0303 10/09/2014). The study flow is depicted in Fig. 1. A genetic counselor/research nurse or PhD student completed the checklist (CCSC, childhood cancer syndrome checklist). The checklist consists of patient characteristics (medical history, tumor type and development), family history and 47 selected specific physical manifestations, which may not be detectable on 2D and 3D photographs of the patient (see Supplementary Appendix 1). The photographs were taken by a medical photographer and consists of a series of 2D photograph of the face (front, portrait, and profile), hands, feet and skin and one 3D photograph of the face. Two independent clinical geneticists (CGs) assessed the checklist and photographs electronically, by means of the decision support scheme. The decision support scheme is a preassembled document, approved by all participating clinical geneticists of the six centers (Supplementary Appendix 2). It states when a patient should be referred to a clinical geneticist for complete genetic consultation. The photographs were used only qualitatively, the CGs viewed the photographs, as if the patient was sitting in front of them. No additional quantitative analyses of facial morphology have been implemented at this point; this is part of a separate study. The clinical geneticists were blinded to each other’s assessment.

Patients in whom one or both clinical geneticists suspected a TPS (i.e., a positive screening result) were referred for complete genetic consultation in the patient’s own treatment center, including molecular analyses when found appropriate by the CG upon routine clinical genetic consultation (‘gold standard’). The CG decided whether and which molecular diagnostics was indicated, based on guidelines for pediatric oncology in the Netherlands [16, 17]. When a clinical case did not match an existing guideline, the CG decided what testing would be appropriate. Patients in whom both clinical geneticists did not suspect a TPS (i.e., a negative screening result) were randomly invited to follow a similar complete genetic consultation as described above.

### Patients

All children diagnosed with cancer in the Netherlands between 01/01/2016 and 31/03/2019 were eligible for the study. Inclusion criteria were age (0–18 years at time of cancer diagnosis), a newly diagnosed malignancy, and written informed consent. Also, patients with benign or borderline malignant tumors, for which treatment by a pediatric oncologist was indicated, were included. Patients were excluded if they had already been identified with a TPS known to be associated with the diagnosed malignancy. In addition, patients with a retinoblastoma were excluded, as genetic consultation in these patients is implemented in routine care in the Netherlands [18]. Routine genetic consultation included molecular studies only if indicated by the clinical genetic consultation mirroring routine genetic practices in the Netherlands.

Characteristics of the participating children were compared with a patient reference population, based on the most recent available national registry data (2015, 2016) from the Dutch Childhood Oncology Group (DCOG).

### Screening tool

The clinical screening tool consists of a checklist, 2D and 3D photographs and a digital assessment of these by a CG. The CGs (n = 8) were guided in their independent assessment by a preassembled decision support scheme, in which nationally agreed reasons for referral were summarized, especially with respect to tumor type (see protocol for details and Supplementary Appendix 2) [14, 17]. For study purposes, all patients were assessed independently by two CGs. The checklist is expert based and consists of a set of open and closed-end (dichotomous, categorical) questions regarding medical history, tumor type, family history and physical examination [15]. The photographic series consists of standardized 2D photographs of face (portrait and profile), hands, feet and skin and a facial 3D photograph.

In variable pairs, individual CGs summarized their assessment of the checklist and photographs digitally. They considered whether referral for a routine genetic consultation (standard care) was indicated. If one or both of the CGs assessed referral was indicated (positive assessment), the patient was referred for routine genetic consultation; this included molecular studies only if indicated by the clinical genetic consultation mirroring routine genetic practices in the Netherlands in the time frame of the study.

Patients in whom referral was not indicated (double negative assessment) were randomly allocated (ratio 3:1, anticipating drop-outs) to no referral or referral for routine genetic consultation, ensuring that at least 20% was evaluated by a CG as a negative control group [15].

### Statistics

Continuous normally distributed variables are expressed by their mean and standard deviation, or when not normally distributed as medians and their interquartile ranges. Categorical variables are expressed as counts (n) and percentages (%). To analyze differences in continuous variables, Student’s *t* test is used, or, in case continuous data are not normally distributed, the Mann–Whitney *U* test is used. Categorical variables are compared with the Chi-square test. A *P* value < 0.05 is considered statistically significant. IBM SPSS statistics V25 was used. Inter-rater reliability was determined calculating Krippendorff's alpha (R-version 3.5.1) and the 95% confidence interval (https://github.com/MikeGruz/kripp.boot).

### Primary and secondary outcome

Primary outcome was the clinical value of the screening tool, expressed as the identification of patients with TPS (sensitivity). Patients with a negative assessment in whom a TPS was diagnosed at genetic consultation were defined false negatives. A TPS was defined as a clinical and/or molecular confirmed diagnosis as concluded by the CG performing the routine genetic consultation, as is standard in the Netherlands.

Secondary outcome was the identification of patients without TPSs (specificity). False positives were patients with positive assessment in whom no TPS was diagnosed at routine genetic consultation. True negatives were defined as patients with a negative assessment, in whom no TPS was diagnosed at routine genetic consultation. True positives were defined as patients with a positive assessment in whom a TPS was confirmed at routine genetic consultation. In addition, we assessed which patients were assessed positive by the CGs and evaluated which parts of the screening tool (checklist, 2D and/or 3D photographic series) were decisive.

## Results

### Flowchart and patient characteristics

Initially the study enrolled patients in the six Dutch pediatric oncology centers, which were all in the process of centralizing care into the single national center (The Princess Máxima Center). This intensive run-up to centralization markedly inhibited screening of all patients for eligibility for the study (Fig. 1). In total 363 patients completed the full screening. Median age of patients at enrollment was 7 years (IQR 3.3–12.8), 54% of patients were male. Baseline patient characteristics were compared to those of the national DCOG registry, and showed no significant differences (Table 1). The distribution of tumor types of study participants differed from those in the DCOG cohort (Table 1 and Supplementary table 1), with a higher proportion of hemato-oncology and a lower proportion of neuro-oncology malignancies in the study cohort. General patient information as collected with the checklist are described in Supplementary Results.

**Table 1:** Characteristics of the present series of children with cancer compared to the data from the national Dutch registry DCOG

	Prospective TuPS cohort n = 363		DCOG reference data^a^ n = 1192
Gender			
Male	197	54%	53%
Female	166	46%	47%
Age at diagnosis in years (median, IQR)	7 (3.3–12.8)		7 (3–13)
Self reported Dutch ethnicity	286	79%	n/a
Self reported consanguinity parents	9	3%	n/a
Tumor types (based on ICCC3)^b^			
** Hemato-oncology**	**186**	**51%**	45%*
Leukemias, myeloproliferative diseases, and myelodysplastic diseases	128	35%	28%
Lymphomas and reticuloendothelial neoplasms	49	14%	13%
Other non ICCC-3	9	3%	5%
** Neuro-oncology**	**38**	**11%**	21%**
CNS and miscellaneous intracranial and intraspinal neoplasms	38	11%	21%
**Solid tumors**	**139**	**38%**	**34%**
Neuroblastoma and other peripheral nervous cell tumors	20	6%	6%
Retinoblastoma	0	0%	2%
Renal tumors	31	9%	5%
Hepatic tumors	2	1%	1%
Malignant bone tumors	25	7%	5%
Soft tissue and other extraosseous sarcomas	42	12%	6%
Germ cell tumors, trophoblastic tumors, and neoplasms of gonads	11	3%	6%
Other malignant epithelial neoplasms and malignant melanomas	7	2%	3%
Other and unspecified malignant neoplasms	1	0%	0%

*n/a* not available

^a^Representing cumulative incidence of the two most recent years (2016, 2017)

^b^For full tumor specification see Supplementary Table 1

^*^Significant difference between TuPS and DCOG cohort (*p* = 0.034)

^**^Significant difference between TuPS and DCOG cohort (*p* = 0.000)

### Assessment by CGs

In 57% (208/363) of the included patients, the assessment of the checklist and photographic series by the CGs was positive and in 43% (155/363) it was negative. The positive assessment was double positive in 116 patients and single positive in 92 patients.

The result of the assessment (checklist and photographs) by two geneticists was unambiguous in 271 patients (75%, “double positive” or “double negative”), and inconclusive (“single positive”) in 25% (n = 92). Patient characteristics of the three assessment groups (double positive, single positive, double negative) did not significantly differ, except for the distribution of the tumor types (Supplementary Table 2). In 47% of the double and 37% of the single positively assessed patients, referral was based on checklist plus photographs (Table 2). In 52% (double positive) and 56% (single positive) group referral was based on the checklist only. Within these latter groups, the tumor types were the primary reason for positive assessment. In 32 patients (32/208, 15%) one (n = 29) or both (n = 3) CGs indicated that the 2D with (12/32) or without (20/32) the 3D photographic series were decisive in their positive assessment. Positive assessment was based solely on physical appearance (physical examination and/or photographic series) in 12% (25/208; solely physical examination n = 6, solely photographic series n = 7, combination n = 12). Thirteen patients (13/208, 6%) were assessed positive solely based on family history.

**Table 2:** Characteristics of children with a defined or potential tumor predisposition syndrome

No	Gender	Age (years)	Tumor	Other findings	Assessment CGs	Assessment based on	Variant	Significance variant	Variant related to tumor
1	F	1	Wilms tumor	Nephrogenic rest other kidney	++	1	c.1-?_646 + ?del in *WT1*	Pathogenic	Yes
2	F	15	Renal cell carcinoma	Cyst other kidney, family history of leiomyomas	++	1, 2, 3, 4	c.-23-?_*148 + ?del in *FH*	Pathogenic	Yes
3	F	3	Wilms tumor		++	1	c.1216_1223del p.(Ser406fs) in *WT1*	Pathogenic	Yes
4	F	8	Optic nerve glioma	Axillary freckling, CAL spots, developmental delay	++	1, 2, 4, 5	c. (?_5206_5546_?) del, p.?) in *NF1*	Pathogenic	Yes
5	M	5	Acute lymphoblastic leukemia	Father with ALL at 9 months of age	++	1, 3	deletion in exon 6 *PAX5*	Unclear	Unknown
6	M	11	High grade glioma (gr III)	Family history of gastric, cervical and breast cancer	++	1, 3	c.2123A > G (p.His708Arg) in *POLE*	Unclear	Unknown
7	F	10	Renal angiomyolipoma		+−	1	c.152A > C (p.Glu51Ala) in *TSC2*	Unclear	Unknown
8	M	7	Inflammatory myofibroblastic tumor of left orbit		+−	1	c.256G > A (p.Asp86Asn) in *CDKN2B* c.1039C > T (p.Gln347*) in *PTCH2*	Unclear	Unknown

*M* male, *F* female, *CAL* café au lait, ++: double positive, +−: single positive, *1* tumor type, *2* medical history, *3* family history, *4* physical examination, *5* 2D pictures, *6* 3D picture

All positively assessed patients were referred to the clinical geneticist for routine consultation (Table 3). In four patients a TPS was diagnosed, in four other patients a variant of uncertain significance was found (Table 4). Thirty-five patients who were assessed negative were randomly allocated into the control group and seen by a clinical geneticist for routine consultation. In the majority of these patients (83%, 29/35) a TPS could be ruled out by the CG without the need for further DNA diagnostics. This was expected, as the two CG’s who performed the screening did not have a suspicion for a TPS (and thus further referral to a CG was deemed not necessary). In six patients (17%, 6/35), the CG ordered DNA diagnostics, which all returned negative. In four of these six patients, molecular studies into constitution mismatch repair syndrome were performed due to hyper/hypo pigmentations of the skin. In one patient with a neurblastoma with a somatic ALK variant, diagnostics were run to see whether this variant could also be found in the germline (negative). The last patient had a Ewing sarcoma, no variant in P53 was identified.

**Table 3:** Rationale for the clinical geneticists to assess a child as having an increased risk for a tumor predisposition syndrome using the TuPS tool

	Double positive	Single positive
	n (%)	n (%)
Number of patients with positive assessment by clinical geneticist	116 (100)	92 (100)
Positive assessment based on^a^		
Checklist	115 (99)	86 (93)
*Specified into*^a^		
Tumor type	83 (72)	36 (39)
Medical history of patient	31 (27)	22 (24)
Family history	57 (49)	30 (33)
Physical examination	37 (32)	34 (37)
2D photographic series	56 (48)	40 (44)
3D photograph	8 (7)	12 (13)

^a^Multiple answers possible

**Table 4:** Results of positively scored children with cancer after routine clinical genetic evaluation and the (negatively scored) control group

	Patients with positive assessment (n = 208)				Patients with negative assessment (n = 155)	
	Double positive (n = 116)		Single positive (n = 92)		Negative control group after randomization (n = 40)	
Patients seen for routine consultation CG^a^	105		81		35	
TPS ruled out without further DNA diagnostics	33	(31%)	56	(69%)	29	(83%)
Further DNA diagnostics						
Offered, but declined	9	(9%)	4	(5%)	0	
Negative	57	(54%)	19	(23%)	6	(17%)
Positive, TPS found	4	(4%)	0		0	
Unclear significance	2	(2%)	2	(3%)	0	

^a^Some patients refrained from further consultation or were unable to visit due to their illness

The observed sensitivity is 100% and the observed specificity is 43% but caution is needed in interpreting these results, as the total number of included patients and the observed prevalence rate of TPS is lower than expected.

### Inter-rater reliability

Eight independent CGs in random pairs of two assessed whether referral was required. The inter-rater reliability for referral, based on 363 patients and 8 raters, determined with the Krippendorff's alpha, was 0.488 (95% CI 0.313–0.657).

### Observed prevalence of TPSs in subset of centralized patients

The centralization of pediatric cancer care in the Netherlands allowed exploring in retrospect the total number of patients with TPSs and the timing of this diagnosis. In the 6 months following centralization, 258 patients were newly diagnosed with cancer (Supplementary Fig. 2). In this group, the prevalence of a TPS was 6% (15/258). Eight children were already known with a TPS at cancer diagnosis. In seven additional patients, a TPS was diagnosed after referral to the CG (Supplementary Table 4). Two of these seven patients were approached for participation in this study, but refused as they had already been evaluated by a CG. The other five patients were not enrolled in the study for unknown reasons.

## Discussion

The primary aim of the present study was to assess the clinical value of a screening tool in identifying childhood cancer patients at risk for a TPS. All children in this study in whom we detected a TPS were assessed at risk for TPS (positive) by the tool. False negatives were not detected in the negative control group seen for routine genetic consultation (n = 35). However, the included study cohort is too small and the observed TPS prevalence lower than expected to allow for a valid conclusion on the accuracy performance of the screening tool. Using the tool, 43% of the children were assessed as not at risk for having a TPS (negative), meaning there was no need for referral to a CG. Optimizing referral may reduce societal costs, while guaranteeing systematic clinical genetic health care for all children with cancer. Furthermore, using the tool results in standardized registration of clinical patient data, which supports routine genetic consultation and can be used in future studies.

We observed a prevalence of TPS of 1% in our cohort (4/363). This low number is mainly explained by the exclusion of patients with a known TPS at the time of diagnosing the cancer, and non-participation of patients who already were in the process of routine genetic consultation at the time of study inclusion. This is reflected in the retrospective analysis of a period of 6 months after the centralization of care, in which 6% of the patients with cancer were found to have an established TPS (Supplementary Results 2). Another important limitation of this study is the number of included patients. We included 363 patients, which is 36% of the expected accrual [15]. The major reasons of this limited accrual are the delay in implementation of this study in the six oncology centers during the process of centralization of pediatric oncology care in the Netherlands. This markedly influenced the ability to identify potential eligible patients. However, the centralization of care and research eventually should make it easier for future studies to be implemented.

Based on lessons learned from the present study, we suggest modifications when implementing the screening tool to increase specificity. Several questions on the score form should be further specified, for instance by specifying the exact number and size of hyperpigmentation. Criteria for referral because of a positive family history and the (molecular) pathology of the tumor can be further specified. Lastly, added value of 3D photographs in the screening tool was not demonstrated in this study. Nevertheless, 2D photography is easily accessible and essential in evaluating a child for a TPS, especially as we see that 12% the children were assessed positive based on physical appearance solely. For research purposes, 3D photography may still be helpful and possibly even indispensable to recognize patterns in facial morphology that are difficult to recognize clinically (Postema et al. submitted). Then 3D scanning is part of research and not of clinical care.

Several decision support tools or suggestions for the referral of children with cancer to the CG have been proposed, none of which have been assessed prospectively [6, 9, 19]. The additional value of our prospectively assessed screening tool compared to the other tools is the explicit use of scoring dysmorphisms and 2D and 3D photographic series. In addition, our screening tool incorporates the knowledge and experience of a CG in the assessment digitally. Lastly, using the tool generates systematically phenotype data, which can be very helpful when interpreting molecular diagnostics. Nevertheless, implementation of this screening tool does require extra efforts; filling out the checklist, taking photographs and their digital assessment. However, the required extra time is considerably less than a consultation by a clinical geneticists. In addition, when a child is referred based on a positive outcome of the screening tool the information already gathered can be incorporated in the genetic consultation, saving time.

In 57% of the patients, one or both CGs assessed patients at risk for TPS (positive). This is comparable to the study by Chan and colleagues, using our checklist and identifying 65% of the patients at risk of a TPS [5]. These numbers are higher compared to other studies, in which a reason for genetic assessment was present in 29–40% of study participants [20, 21]. This difference may partly be explained by our use of photographs and specified physical examination as this allowed identifying an additional 25 patients (of 208; 12%) at risk for a TPS, as these were only recognized based on one of these items.

In 92 patients (25%) the assessment of checklist and photographs by the geneticists as part of the tool was not unequivocal. We were unable to determine whether CG characteristics (experience or other factors) influenced this. The modest Krippendorf’s alpha for referral suggests that a measurement error/bias was introduced by the independent CGs.

The approach in the diagnostics of TPSs is shifting, from first clinical evaluation and subsequent targeted molecular testing when indicated, to a sequencing first approach. For the foreseeable future, the gold standard will be a combination of both. Next generation sequencing can identify variants in genes known or expected to cause TPSs, but clinical information and evaluation by a geneticist are essential to determine the relevance of such variants.. Clinical evaluation will therefore remain essential. Comparable to the routine practice at the time we designed the study, molecular studies were not a standard part of the present study. Future research should establish the optimal balance between using clinical and genomic data in identifying and reliably diagnosing TPSs in childhood cancer patients [22].

## Conclusion

This is the first prospective study assessing the clinical validity of a screening tool for TPSs in childhood cancer patients, including detailed physical characteristics. In our cohort we found a prevalence of TPSs of 1%. This lower than expected prevalence is explained by important inclusion bias, in part explained by the design of the study where patients already known with a TPS were excluded for obvious reasons. All children with a diagnosed TPS in the study were assessed positive using the tool. Using the tool, 43% of the newly diagnosed children with cancer were assessed not at risk for having a TPS. Adaptations of the tool may increase specificity without decreasing sensitivity. The tool ensures a systematic collection of clinical and morphology data needed for interpretation of genetic assessment in a patient.

## Supplementary Information

Below is the link to the electronic supplementary material.

Supplementary file 1 (PDF 1545 KB)

## Data Availability

Data available upon request.
