# Supplementary material for: Clinical value of a screening tool for tumor predisposition syndromes in childhood cancer patients (TuPS): a prospective, observational, multi-center study
Source: Fam Cancer. 2021 Mar 9;20(4):263–71. doi: 10.1007/s10689-021-00237-1 (PMC8484098; doi:10.1007/s10689-021-00237-1)
Supplement: Supplementary file 1 — (PDF 1545 KB) [file 10689_2021_237_MOESM1_ESM.pdf]

Supplementary Material

Clinical Value of a Screening Tool for Tumor Predisposition Syndromes in Childhood Cancer Patients

Postema FAM et al.

Index

Supplementary Results page 2

General patient information collected with the checklist

Supplementary Figure 1. page 3

Reported history of cancer in families of study participants

Supplementary Figure 2. page 4

Prevalence study in subset of centralized patients with cancer in the first 6 months after opening of the Princess Maxima Center

Supplementary Table 1. page 5

Tumor specification of study participants according to ICCC3 classification

Supplementary Table 2. page 7

Characteristics of study participants subdivided by the scoring of the clinical geneticists using the TuPS tool

Supplementary Table 3. page 8

Abnormalities detected at physical examination using the CCSC as part of the screening tool

Supplementary Table 4. page 9

Characteristics of children with a tumor predisposition syndrome, referred outside the study to the clinical geneticist, during 6 months in the national pediatric oncology center (Princess Maxima Center)

Supplementary Appendix 1. page 10

Childhood Cancer Syndrome Checklist

Supplementary Appendix 2. page 13

Decision support scheme

Supplementary Results

General patient information collected with the checklist

Based on the information registered in the checklist, we observed one patient (0.3%) having a tumor (choroid plexus papilloma) prior to his current malignancy. In 20 patients (5.5%) motor development and in 13 (3.6%) cognitive development was delayed. Puberty was advanced in three patients (0.8%) and delayed in one patient (0.3%). A family member (up to and including the third degree) with a history of cancer was present in 81% (Supplementary Figure 2). In 228 patients (63%), no morphological abnormalities were found at physical examination. Hyperpigmentation (one or more spots) was present in 109 patients (30%). The assessing CG determines whether that was a reason for referral or not. Fifty-two of these children (52/109, 48%) were assessed positive by at least one CG based on their physical examination (including but not limited to hyperpigmentation), however, almost always in combination with other items such as tumor type (49/52, 94%). A list of all registered morphological abnormalities is presented in Supplementary Table 3.

Supplementary Figure 1. Reported history of cancer in families of study participants

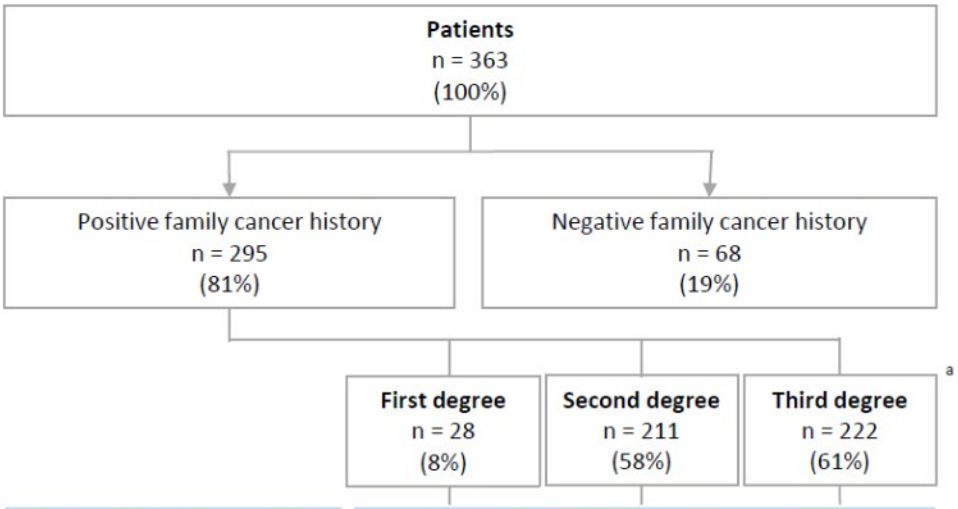

Number of family members with cancer	Number of children with cancer with [x] family members with cancer		
x = 1	27 (96%)	130 (62%)	93 (42%)
x = 2		60 (28%)	52 (23%)
x = 3	1 (4%)	13 (6%)	34 (15%)
x >3		8 (4%)	43 (19%)

Age category of family members with cancer	Number of children with cancer with one or more family members in this age category[b]		
0-18	3 (10%)	6 (3%)	10 (5%)
19-45	18 (64%)	48 (23%)	60 (27%)
46+	6 (21%)	172 (82%)	172 (77%)
unknown	1 (4%)	25 (12%)	45 (20%)

[a] Adds up to > 81%, as a patient can have several family members with cancer
[b] Adds up to > 100%, as one patient can have several family members with cancer

Supplementary Figure 2. Prevalence study in subset of centralized patients with cancer in the first 6 months after opening of the Princess Maxima Center

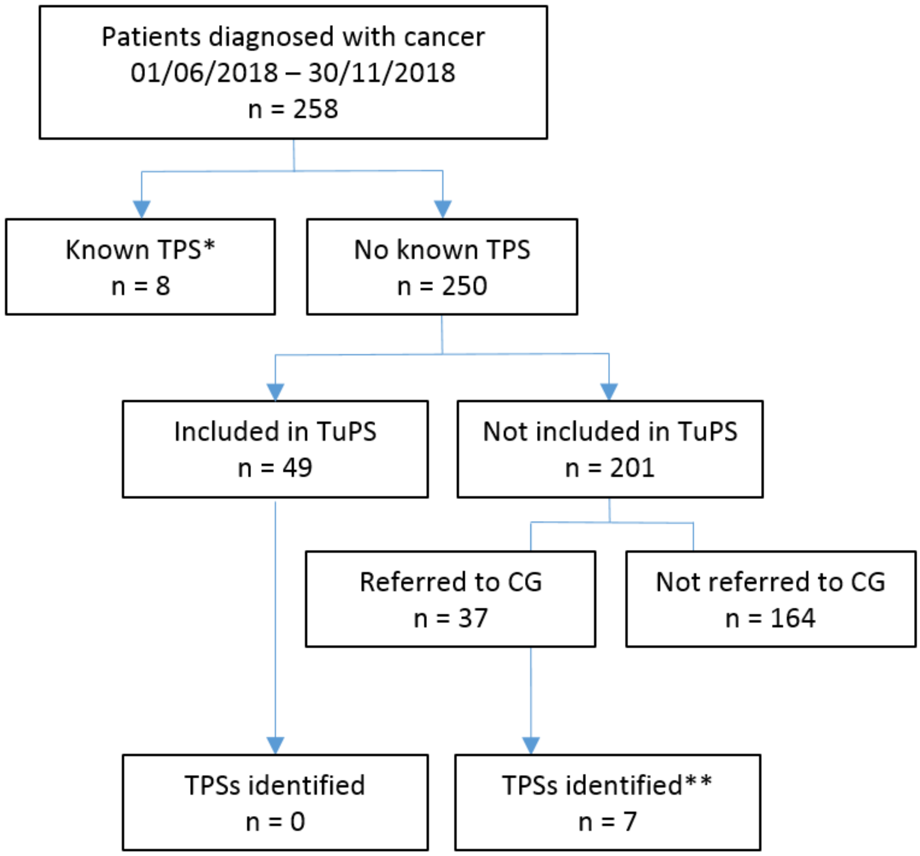

* Congenital hypoventilation syndrome (n = 1), Neurofibromatosis type 1 (n = 1), Neurofibromatosis type 2 (n = 1), Shwachman-Diamond syndrome (n = 1), Down syndrome (n = 4)

** *WT1* mutation (n = 3), *TP53* mutation (n = 2), *REST* mutation (n = 1), Beckwith-Wiedemann syndrome (n = 1), see Supplementary Table 4

Supplementary Table 1. Tumor specification of study participants according to ICCC3 classification

	**Prospective TuPS cohort**	
	**n = 363**	**%**
**Hemato-oncology**	186	51.0
1. Leukemia's, myeloproliferative diseases, and myelodysplastic diseases	128	35.3
Lymphoid leukemia's	110	30.3
Acute myeloid leukemia's;	15	4.1
Chronic myeloproliferative diseases	3	0.8
Myelodysplastic syndrome and other myeloproliferative diseases	0	0.0
Unspecified	0	0.0
2. Lymphomas and reticuloendothelial neoplasms	49	13.5
Hodgkin lymphoma	22	6.1
Non-Hodgkin lymphoma (excl Burkitt lymphoma)	19	5.2
Burkitt lymphoma	7	1.9
Lymphoreticular malignancy	0	0.0
Unspecified	1	0.3
Other non ICCC-3	9	2.5
Fanconi anemia	0	0.0
Langerhans Cell Histiocytosis	9	2.5
**Neuro-oncology**	38	10.5
3. CNS and miscellaneous intracranial and intraspinal neoplasms	38	10.5
Ependymoma, choroid plexus carincoma	5	1.4
Astrocytoma, glioblastoma, optic nerve glioma	12	3.3
Intracranial and intraspinal embryonal tumors	13	3.6
Other gliomas	5	1.4
Other specified intracranial and intraspinal neoplasm	3	0.8
Unspecified	0	0.0
**Solid tumors**	139	38.3
4. Neuroblastoma and other peripheral nervous cell tumors	20	5.5
Neuroblastoma and ganglioneuroblastoma	20	5.5
Other peripheral nervous cell tumors	0	0.0
5. Retinoblastoma	0	0.0
Retinoblastoma	0	0.0
6. Renal tumors	31	8.5
Wilms tumor	30	8.3
Renal carcinoma	1	0.3
7. Hepatic tumors	2	0.6
Hepatoblastoma	2	0.6
Hepatocellular carcinoma	0	0.0
8. Malignant bone tumors	25	6.9
Osteosarcoma	11	3.0
Chondrosarcoma	0	0.0
Ewing sarcoma	14	3.9
Other specified malignant bone tumor	0	0.0
Unspecified	0	0.0
9. Soft tissue and other extra-osseous sarcomas	42	11.6
Rhabdomyosarcoma	28	7.7
Fibroblastic and myofibroblastic tumor, peripheral nerve sheath tumor	8	2.2
Kaposi sarcoma	0	0.0
Other specified	5	1.4

Unspecified	1	0.3
10. Germ cell tumors, trophoblastic tumors, and neoplasms of gonads	11	3.0
Intracranial and intraspinal germ cell tumors	2	0.6
Malignant extracranial and extra-gonadal germ cell tumors	4	1.1
Malignant gonadal germ cell tumors	5	1.4
Gonadal carcinoma	0	0.0
Other	0	0.0
11. Other malignant epithelial neoplasms and malignant melanomas	7	1.9
Adrenocortical carcinoma	0	0.0
Thyroid carcinoma	0	0.0
Nasopharyngeal carcinoma	1	0.3
Malignant melanoma	3	0.8
Skin carcinoma	1	0.3
Other	2	0.6
12. Other and unspecified malignant neoplasms	1	0.3
Other malignancies	1	0.3
Not specified	0	0.0

Supplementary table 2. Characteristics of study participants subdivided by the scoring of the clinical geneticists using the TuPS tool

	Double positive	*Single positive*	*Double negative*
	n = 116	*n = 92*	*n = 155*
Gender (%)			
Male	56	46	58
Female	44	54	42
Age at diagnosis in years (median, IQR)	5.9 (2.8-12.5)	6.9 (3.5-12.8)	7.8 (3.5-12.9)
Self-reported Dutch ethnicity (%)	80	80	77
Self-reported consanguinity parents (%)	3	3	2
Tumor types (based on ICCC3)*			
Hemato-oncology (%)	30	55	65
Neuro-oncology (%)	16	11	7
Solid tumors (%)	54	34	29

* Significant difference between assessment groups with *P* = 0.000 using Chi-square test

Supplementary Table 3. Abnormalities detected at physical examination using the CCSC as part of the screening tool

	%	Number of patients scored positive / Number of patients evaluated
Hyperpigmentation	30.0	109/353
Tongue abnormality	1.9	7/353
Body asymmetry	1.7	6/357
Umbilical hernia	1.7	6/351
Scalp tumors	1.4	5/359
Photosensitivity	1.4	5/356
Skin tumors	1.4	5/354
Crease/pits of ear lobule	1.1	4/360
Axillary freckling	1.1	4/353
Supernumerary nipple	1.1	4/351
Leukoplakia	0.8	3/354
Thin skin	0.6	2/355
Oral pigmentation	0.6	2/353
Helical pits of ear helix	0.3	1/360
Cranial nerve palsy	0.3	1/347
Ataxia	0.3	1/339
Cataract	0.0	0/361
Brittle hair	0.0	0/350
Visible nerve fibers on cornea	0.0	0/361
Palmar pits	0.0	0/360
Telangiectasia	0.0	0/354
Blue nevus	0.0	0/354
Oral tumors	0.0	0/353
Abnormal oral mucosa	0.0	0/353
Mucosal neurinomas	0.0	0/352
Peri-orificial papilloma	0.0	0/352
Enlarged thyroid	0.0	0/351
Ambiguous genitalia	0.0	0/218
Abnormal genital pigmentation	0.0	0/216

Supplementary Table 4. Characteristics of children with a tumor predisposition syndrome who not participated in the TuPS study. These patients were referred to the clinical geneticist by their pediatric oncologists during the first 6 months after opening of the national pediatric oncology center (Princess Máxima Center).

Age at diagnosis (years)	Gender	Referred to CG based on	Tumor	Variant
1.4	Male	Tumor, medical history	Wilms tumor	c.457G>T p.(Glu153*) in *WT1*
0.8	Male	Tumor	Wilms tumor	c.1223_1225delinsAAAG p.(Leuk408*) in *WT1*
2.6	Female	Family history	Rhabdomyosarcoma	c.623A>T p.(Asp208Val) in *TP53*
12.1	Female	Tumor in history	Acute lymphoblastic leukemia	c.800G>C in *TP53*
1.3	Female	Tumor, family history	Bilateral Wilms tumor	c.843del p.(Cys281*) in *REST*
4.8	Male	Tumor	Wilms tumor	Hypermethylation H19/IGF2 chromosome 11p15
0.6	Female	Tumor	Wilms tumor	c.1213_1214del p.(Lys405fs) in *WT1*

During the six months period, a study into the genotypic characterization of Wilms tumors was running. Children with a Wilms tumor included in this study underwent extensive genetic analysis into the existence of a predisposition.

Supplementary Appendix 1: Childhood Cancer Syndrome Checklist

TuPS

Please note that this checklist is part of a screening instrument which has been subject to an initial, limited validation. Prudence is called for preliminary use in clinical practice

SCREENING INSTRUMENT TUMOUR PREDISPOSITION SYNDROMES IN CHILDHOOD CANCER PATIENTS

Part I: Childhood cancer syndrome checklist (CCSC)

Version may 2016

EXCLUSION CRITERIA

Already diagnosed with a syndrome known to be associated with the current observed malignancy. No/yes

* see table 1: syndromes and tumours/leukaemia's (instruction form)

INCLUSION CRITERIA

Age 0-18 years	Yes/No
Newly diagnosed tumour or leukaemia	Yes/No
Signed informed consent by both parents/guardians	Yes/No
Signed informed consent by patient	Yes/No/Not applicable (<12years)
Date informed consent	|__|__| |__|__|__| |__|__|__|__|

NB. If the criteria are not met, participation in the study stops here. There is no need in entering data in the online database OpenClinica.

PATIENT CHARACTERISTICS

Study number: |__|__|__|__|__|__|__|

Month of birth: |__|__|__|

Year of birth: |__|__|__|__|

Sex: m/f

Ethnic background:
- o Dutch
- o Turkish
- o Moroccan
- o Hindu
- o Unknown
- o Other, i.e.______________________________

Oncologic diagnosis: _______________________

Anatomic localisation: ______________________

Month of diagnosis: |__|__|__|

Year of diagnosis: |__|__|__|__|

Therapy started: yes/no

If so;
- ☐ Chemotherapy
- ☐ Surgery
- ☐ Radiotherapy

Specify: _____________________________

Previously tumour/leukaemia: yes/no

(Both benign and malignant)

If so;

*Specify:*________________________________

*Treatment:*____________________________

Pregnancy

Term labour |__|__|+|__| weeks + days

Weight at birth |__|__|__|__| gram

Complications: yes/no

If so, which? __________________________

Development

Motor:
- o Normal
- o Late

Cognition:
- o Normal
- o Late

Puberty:
- o Normal
- o Not applicable yet
- o Early
- o Late

Other medical history:

(hospital admissions, contacts medical specialist, congenital organ defects)

____

____

____

____

Please, store this checklist carefully.

FAMILY HISTORY
NB. Half-siblings are counted as full siblings

Table 1. Ask for the following aspects

**Who?**	**Tumour/leukaemia + age of diagnosis**	**Morphological abnormalities/ Congenital anomalies**	**Learning- and developmental difficulties**
We use the following nomenclature:<br>**M**= mother (mater)<br>**P**= father (pater)<br>**F**= brother (frater)<br>**S**= sister (soror)<br>**Fs** = son (filius)<br>**Fe**= daughter (filia)<br>*For example for a cousin who is the son of the brother of mother: FsFM*	Both benign and malignant tumours (including cysts)<br><br>*+ type (which tumour)*<br>*+ localisation (what body part)*<br>*+ age of diagnosis*	Congenital anomalies which stand out in appearance, where a person is operated on, where someone receives treatment for, or is limited by.<br><br>*+ type (which anomaly)*<br>*+ localisation (what body part)*	Learning difficulties, developmental delay, intellectual disabilities or behavioural problems for which extra support is necessary.<br><br>+ type (which difficulty/delay/problem)<br>+ degree of support

Table 2. Please use the items listed in Table 1 when filling in the following table.

Who?	***What?*** *(+ age)*		***Who?***	***What?*** *(+ age)*

Are parents related? No/Yes

siblings
Number of brothers: |__|__|
Number of sisters: |__|__|
Are there any particularities with the brothers or sisters? No/Yes
If so, please fill in table 2.

parents
Number of brothers of father: |__|__| Number of brothers of mother: |__|__|
Number of sisters of father: |__|__| Number of sisters of mothers: |__|__|
Are there any particularities with the father or mother? No/Yes
If so, please fill in table 2.

uncles & aunts
Are there any particularities with the uncles or aunts? No/Yes
If so, please fill in table 2.

cousins
Are there any particularities with the cousins? No/Yes
If so, please fill in table 2.

grandparents
Father's side
Number of sibs grandfather: |__|__|
Number of sibs grandmother: |__|__|
Mother's side
Number of sibs grandfather: |__|__|
Number of sibs grandmother: |__|__|
Are there any particularities with the grandfathers and grandmothers and their siblings?
If so, please fill in table 2.

Are there relatives of whom you don't have general information about their health? No/Yes
If so, who are they?
____

PHYSICAL EXAMINATION Date of examination: |__|__| |__|__|__| |__|__|__|__|

ANTHROPOMETRICS
Height: |__|__|__|cm Weight: |__|__|.|__| kg Head circumference : |__|__|.|__|cm

Please use the booklet **Tumour Predisposition syndrome in Childhood Cancer Screening Instrument, part II: Definitions and pictures** when filling in this list. For items with a * there are illustrating pictures available.

HEAD

Cranium

Scalp tumours	no/yes/unable to evaluate/not evaluated
Brittle hair*	no/yes/unable to evaluate/not evaluated

Eyes

Cataract*	no/yes/unable to evaluate/not evaluated
Visible nerve fibres on cornea*	no/yes/unable to evaluate/not evaluated
Photosensitivity*	no/yes/unable to evaluate/not evaluated
	If so ☐ eyes ☐ skin

Ears

Crease/pits of ear lobule*	no/yes/unable to evaluate/not evaluated
(Posterior) helical pits of ear helix*	no/yes/unable to evaluate/not evaluated

Mouth/oral region

Leukoplakia*	no/yes/unable to evaluate/not evaluated
Tongue*	normal/abnormal/unable to evaluate/not evaluated
	If abnormal ☐ large ☐ lobulated ☐ protruding
Oral pigmentation*	no/yes/unable to evaluate/not evaluated
Oral tumours	no/yes/unable to evaluate/not evaluated
Abnormal oral mucosa (cobblestone)*	no/yes/unable to evaluate/not evaluated
Mucosal neurinomas*	no/yes/unable to evaluate/not evaluated
Papilloma peri-orificial*	no/yes/unable to evaluate/not evaluated

THORAX

Supernumerary nipples*	no/yes/unable to evaluate/not evaluated

ABDOMEN

Umbilical hernia*	no/yes/unable to evaluate/not evaluated

EXTRIMITIES

Asymmetry (length, width, both)	no/yes/unable to evaluate/not evaluated
	If so ☐ arms ☐ legs
Palmar pits	no/yes/unable to evaluate/not evaluated

GENETALIA

Abnormal genital pigmentation*	no/yes/unable to evaluate/not evaluated
Ambiguous genitalia*	no/yes/unable to evaluate/not evaluated

SKIN

Telangiectasia*	no/yes/unable to evaluate/not evaluated
	If so ☐ conjunctival ☐ nasal ☐ oral
Tumours*	no/yes/unable to evaluate/not evaluated
Blue naevus*	no/yes/unable to evaluate/not evaluated
Axillary freckling* `	no/yes/unable to evaluate/not evaluated
Hyperpigmentation*	no/yes/unable to evaluate/not evaluated
Thin skin/generalized skin atrophy*	no/yes/unable to evaluate/not evaluated

NEUROLOGICAL

Ataxia*	no/yes/unable to evaluate/not evaluated
	If so ☐1☐2
Cranial nerve palsy*	no/yes/unable to evaluate/not evaluated
	If so ☐1☐2☐3☐4☐5☐6☐7☐8☐9

ENDOCRINE

Enlarged thyroid*	no/yes/unable to evaluate/not evaluated

Correctly completed and verified by: |__|__|

Please, store this checklist carefully.

Supplementary Appendix 2: Decision support scheme

Version May 2016

Validation screening instrument tumour predisposition syndromes in childhood cancer patients

DECISION SUPPORT SCHEME

For clinical geneticists assessing the childhood cancer syndrome checklist and the 2D and 3D photographic series.

Referral to the clinical genetics department follows when there is suspicion for a tumour predisposition syndrome (TPS), based on one or more of the following items;

1. Tumour

NB. This list is not comprehensive. You are free to refer every patient to the clinical geneticist if there is any suspicion of a TPS based on the tumour; for example a very rare tumour, a specific malignancy on a unsuspected age or on an unusual location.

- Adrenocortical carcinoma
- Aggressive fibromatosis
- Atypical teratoid/rhabdoid tumour
- Choroid plexus carcinoma
- Endolymphatic sac tumour
- Gangliocytoma
- Hemagioblastoma
- Hepatoblastoma
- Hepatocellular carcinoma
- Juvenile myelomonocytic leukaemia
- Medulloblastoma
  not if known non-SHH subtype and the child is >3 years old.
- Meningioma
- Myelodysplastic syndrome
- Nephroblastoma (Wilms tumour)
- Optic nerve glioma
- Peripheral nerve sheath tumour
- Pineoblastoma
- Pituitary adenoma
- Pituitary blastoma
- Pleuropulmonary blastoma
- Renal carcinoma
- Retinoblastoma
- Rhabdomyosarcoma
  not if the child is >3 years old.
- Spinal ependymoma
- Skin carcinoma
- Subependymal giant cell astrocytoma
- Thyroid carcinoma
- Vestibular schwannoma
- Adult tumours; e.g. carcinoma, pheochromocytoma

2. History

- Prior primary malignancy
- Perinatal data, learning and developmental difficulties, or growth failure possible existing in the context of a TPS
- Other medical issues possible existing in the context of a TPS

3. Family history *(see figure 1)*

NB. This list is not comprehensive. You are free to refer every patient to the clinical geneticist if there is any suspicion of a TPS based on family history, for example consanguinity of parents and a specific tumour. The following situations will often be a reason for referral of the patient to the clinical geneticist:

- ≥2 times the presence of the same specific kind of cancer (on one side of the family till the 3th degree) which could be associated with the malignancy of the child.
- Another family member with childhood cancer (≤18 years) which could be associated with the malignancy of the child.
- ≥2 family members (on one side of the family till the 3th degree) with cancer <45 years of age, which could be associated with the malignancy of the child.
- A first degree family member of this child (parent, sibling) with cancer has (had) cancer their self.

4. Morphological examination

Abnormalities in the appearance suggestive for a TPS.

- Found during physical exam (checklist)
- 2D photographic series
- 3D photograph

TuPS

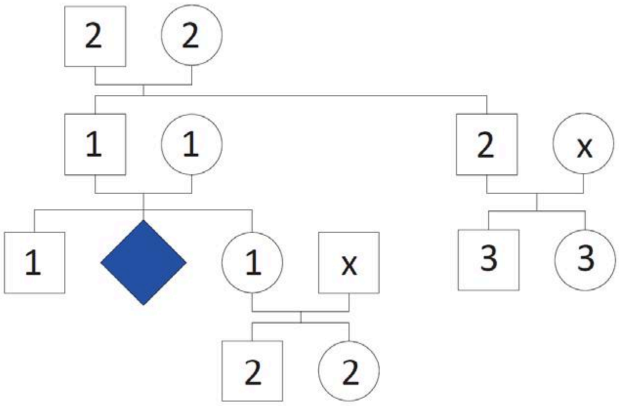

Figure 1. Degree of family members

First degree family members: parents, children, siblings. Second degree family members: siblings of parents, children of siblings. Third degree family members: children of siblings of parents. NB. Half-siblings will be counted as full siblings
